# Oncological and functional outcomes of open versus laparoscopic partial nephrectomy in T1b tumors: A single-center analysis

**DOI:** 10.1590/S1677-5538.IBJU.2018.0865

**Published:** 2020-02-20

**Authors:** Ibrahim Kartal, Nihat Karakoyunlu, Çağlar Çakici, Osman Karabacak, Levent Sağnak, Hamit Ersoy

**Affiliations:** 1 University of Health Sciences Dişkapi Yildirim Beyazit Training and Research Hospital Department of Urology Ankara Turkey Department of Urology, University of Health Sciences, Dişkapi Yildirim Beyazit Training and Research Hospital, Ankara, Turkey

**Keywords:** Carcinoma, Renal Cell, Warm Ischemia, Disease-Free Survival

## Abstract

**Purpose:**

This study aims to evaluate the oncological and functional results of open partial nephrectomy (OPN) and laparoscopic partial nephrectomy (LPN) at the T1b clinical stage, which constitutes 25% of renal cell carcinomas (RCC) at diagnosis.

**Materials and Methods:**

The characteristics of 63 patients with stage T1b solitary tumor who underwent OPN (41) or LPN (22) were compared. The survival analysis was performed using the Kaplan-Meier method. Univariate and multivariate Cox regression analyses were performed to determine the factors affecting disease-free survival. Potential predictive factors, which might affect the postoperative glomerular filtration rate (GFR), were evaluated using multivariate linear regression analysis.

**Results:**

No differences were observed between OPN and LPN groups regarding patient and tumor characteristics. Although the warm ischemia time, intraoperative estimated blood loss, and operation duration were higher in the LPN group, no differences were noted between the two techniques regarding complication rates (p<0.001, p=0.023, p≤0.001, and p=0.190, respectively). The median hospitalization time was shorter in the LPN group than that in the OPN group (4 and 5 days, respectively), with less severe complications. No intergroup differences were observed regarding cancer-specific survival (CSS), disease-free survival (DFS), and overall survival (OS). The evaluation of the factors affecting DFS showed that age was an effective parameter (RR = 1.112, 95% CI: 1.010–8.254), but the surgical technique was not.

**Conclusion:**

No differences were observed between OPN and LPN techniques between oncological and functional outcomes in patients with clinical stage T1b RCC.

## INTRODUCTION

Renal cell carcinoma (RCC) is one of the most common malignancies among genitourinary cancers, detected at an early (localized) stage based on the increased incidental diagnosis, and >70% patients are at stage T1. The optimal treatment of localized RCC is surgery (1). Despite satisfactory oncological results of radical nephrectomy for localized RCC, risk was reportedly higher in patients with chronic kidney disease. Therefore, the preservation of renal parenchyma is recommended for stage-T1 tumors to reduce morbidity (2, 3). Compared with radical nephrectomy, partial nephrectomy (PN) provided better preservation of the renal function and similar oncological outcomes, and therefore, it became the standard treatment for T1 tumors, especially stage T1a, per the guidelines (4, 5).

Despite OPN being the sought-after standard treatment of T1 tumors, technological development and increased preference for minimally-invasive procedures have led to the popularity of the conventional and robot-assisted laparoscopic partial nephrectomy (LPN) in T1 tumors. LPN is generally preferred for T1a tumors. However, >25% RCC cases are determined in the clinical stage T1b (6). Clinicians are using LPN apprehensively for T1b tumors even in cases with an increased tumor size, which may negatively affect the oncological outcomes.

Based on our experience regarding the endourological methods, we evaluated the difference between OPN and conventional LPN in terms of their oncological and functional results in T1b tumors.

## MATERIALS AND METHODS

We evaluated the data of 63 patients, who were initially diagnosed with RCC and at clinical stage T1b and underwent OPN (n=41) or LPN (n=22) in our clinic between January 2012 and June 2015. Only patients with solitary tumors were included. Patients with synchronous bilateral, metachronous, multiple ipsilateral tumors, distant metastasis, and hereditary RCC syndrome were excluded. The longest tumor diameter observed in the imaging method was accepted as the tumor size. After the patients were informed, the surgical approach was chosen based on the surgeon’s experience and opinion regarding the surgical applicability. Three experienced endourologists performed the laparoscopic interventions. The demographic, intraoperative, and postoperative information were extracted from the designed and updated data.

The renal tumor complexity was calculated using R.E.N.A.L. nephrometry scoring system (radius, exophytic/endophytic, nearness of tumor to collecting system, anterior/posterior, hilar tumor touching main renal artery or vein, and location relative to polar lines) (7).

In both procedures, after the dissection of the perinephric fat tissue following the retainment of the fat tissue only on the tumor, the renal artery and vein were separately clamped as far as possible. The tumor was excised with cold knife and sharp incision after considering a safety margin around the tumor and leaving the renal parenchyma intact. Following the closure of the tumor base and parenchyma, the clamps were quickly released. The adjuvant hemostatic agents were used per the surgeon’s preference. The complications were classified based on the modified Clavien classification (8).

The follow-up was performed every 3 months in the first year, every 6 months in the second and third years, and yearly thereon. Abdominal computed tomography was performed at each visit. Magnetic resonance imaging was performed in patients with renal failure or hypersensitivity to contrast agents. DFS, CSS, and OS analyses were performed using the Kaplan-Meier method for each technique followed by an inter-technique comparison. The effects of all risk factors expected to be effective on DFS were evaluated using uni- and multivariate Cox proportional hazard regression method.

Estimated glomerular filtration rate (GFR) and modification of diet in renal disease (MDRD) for each patient were calculated per the equation [eGFR in mL/minute/1.73m^2^=186.3 x (serum creatinine)^−154^× (age)^−0203^ × (0.742 if female) × (1.212 if black)] (9). The GFR values in the preoperative and postoperative periods (first day, the sixth month, and last visit) were compared. The effects of all potential factors (age, ASA, ischemia time, surgical procedure) on surgery, which may be predictive in the estimation of GFR changes (ΔGFR), were investigated using multivariate linear regression analysis in the sixth postoperative month and at the last visit and compared with the preoperative level.

### Statistical analysis

Normal and non-normal distributions of continuous variables were evaluated using the Kolmogorov-Smirnov test. Levene’s test was used to assess the homogeneity of variances.

The mean intergroup differences were compared using the Student’s t-test, and the Mann-Whitney U test was applied to compare the data with non-normal distribution. The categorical data were analyzed using the continuity corrected Chi-square or Fisher’s exact test, where appropriate.

The significance of the correlations between patient characteristics and DFS was assessed using the univariate Cox’s proportional hazard regression analyses. The best predictor(s) of DFS were evaluated using the multivariate Cox’s proportional hazard regression analysis. The relative risk and 95% confidence intervals were also calculated for each independent variable. DFS, OS, and CSS rates were calculated using the Kaplan-Meier survival analysis, and the surgical techniques were compared using the log-rank test. The 5-year cumulative survival rates with a confidence interval of 95% were calculated for each surgical technique.

The best predictor(s) of ΔGFR were evaluated using the multivariate Cox’s proportional hazard regression analysis. Coefficients of regression and 95% confidence interval were also calculated for each independent variable.

Data analysis was performed using IBM SPSS Statistics version 17.0 software (IBM Corporation, Armonk, NY, USA). A p value of less than 0.05 was considered statistically significant. However, Bonferroni correction was applied for all possible multiple comparisons to control type I error.

## RESULTS

No statistically significant differences were noted between the OPN and LPN groups, treated at the clinical stage T1b and concordant with the criteria regarding the mean age, gender distribution, localization, mean BMI, ASA score, median tumor size, and median R.E.N.A.L. score (p>0.05; Table-1).

**Table 1:** Preoperative characteristics of patients and tumors according to surgical procedures.

		OPN (n = 41)	LPN (n = 22)	p-value
**Age** (year), mean		58.2 ± 10.7	52.7 ± 11.1	0.061^a^
**Gender** (n), %				0.999^b^
	Male	25 (61.0%)	14 (63.6%)	
	Female	16 (39.0%)	8 (36.4%)	
**Side** (n), %				0.740^b^
	Right	21 (51.2%)	13 (59.1%)	
	Left	20 (48.8%)	9 (40.9%)	
**BMI** (kg/m^2^), mean		27.5 ± 3.3	25.9 ± 3.0	0.064^a^
**ASA score** (n), %				0.205^b^
	I-II	28 (68.3%)	19 (86.4%)	
	III-IV	13 (31.7%)	3 (13.6%)	
**Tumor size** (mm), median		51.0 (41.0–74.0)	47.5 (42.0–75.0)	0.236^c^
**R.E.N.A.L score** (median)		8.0 (5.0–11.0)	8.0 (6.0–10.0)	0.188^c^

**OPN** = Open partial nephrectomy; **LPN** = Laparoscopic partial nephrectomy; **BMI** = Body mass index; **ASA** = American Society of Anesthesiologists; **R.E.N.A.L** = Radius, exophytic/endophytic, nearness of tumor to collecting system, anterior/posterior, hilar tumor touching main renal artery or vein and location relative to polar lines);

a = Student’s t-test;

b = Continuity Corrected Chi-square test;

c = Mann-Whitney U test.

The median values of WIT, estimated blood loss, and operation duration were significantly higher in the LPN group than in the OPN group (p<0.001, p=0.023, p<0.001, respectively).

No significant intergroup differences were observed regarding intraoperative erythrocyte suspension transfusion, operation duration, hospitalization time, postoperative complication rate, grade distribution among the patients with complication, pathological assessment, Fuhrman nuclear grade, positive surgical margin, follow-up time, and mortality (p>0.05). Regarding the intraoperative complications, a pleural injury occurred in two patients who underwent OPN treated with primary suturing during the operation (Table-2).

**Table 2:** Perioperative and postoperative results according to the surgical technique.

		OPN (n = 41)	LPN (n = 22)	p-value
**Warm ischemia time** (min), median		16.0 (5.0–27.0)	25.5 (5.0-60.0)	**<0.001**^a^
**Estimated blood loss** (mL), median		250 (100-1850)	400 (100-1200)	**0.023**^a^
**Intraoperative ES transfusion,** (pack), median		0 (0-4)	0 (0-6)	0.112^a^
**Duration of operation** (min), median		120 (60-180)	155 (90-240)	**<0.001**^a^
**Hospitalization time** (day), median		5 (2-16)	4 (3-7)	0.221^a^
**Intraoperative pleural injury** (n), %		2 (4.9%)	0 (0.0%)	0.538^b^
**Postoperative complications** (n), %		6 (14.6%)	7 (31.8%)	0.190^b^
**Postoperative complications** (n), %				0.103^b^
	Grade < 3	1 (16.7%)	5 (71.4%)	
	Grade ≥ 3	5 (83.3%)	2 (28.6%)	
**Wound site infection**		1 (2.4%)	1 (4.5%)	0.999^b^
**Urine leakage**		3 (7.3%)	3 (13.6%)	0.413^b^
**Blood transfusion**		0 (0.0%)	2 (9.1%)	0.118^b^
**Prolonged ileus**		0 (0.0%)	1 (4.5%)	0.349^b^
**Re-operation due to bleeding**		2 (4.9%)	0 (0.0%)	0.538^b^
**Pathologic evaluation** (n), %				
	Benign	3 (7.3%)	3 (13.6%)	0.413^b^
	Clear Cell Ca	31 (75.6%)	17 (77.3%)	0.999^c^
	Non-Clear Cell Ca	7 (17.1%)	2 (9.1%)	0.476^b^
**Fuhrman nuclear grade** (n), %				0.478^b^
	Grade I-II	32 (84.2%)	14 (73.7%)	
	Grade III-IV	6 (15.8%)	5 (26.3%)	
**Surgical margin positivity** (n), %		2 (4.9%)	2 (9.1%)	0.606^b^
**Follow-up time** (month), median		54 (37-78)	62 (27-78)	0.471^a^
**Death** (n), %		4 (9.8%)	2 (9.1%)	0.999^b^
	Oncological	2 (4.9%)	1 (4.5%)	0.999^b^
	Non-oncological	2 (4.9%)	1 (4.5%)	0.999^b^

a = Mann-Whitney U test;

b = Fisher’s exact test;

c = Continuity Corrected Chi-square test;

ES = Erythrocyte suspension.

All treatment modalities were successfully applied during the perioperative period, and no operation-associated mortality was observed. Six complications occurred in OPN group (14.6%) and seven in the LPN group (31.8%). Despite the lower complication rate in the OPN group, major complications (grade 3) were observed in five patients (two were re-operated owing to bleeding).

In the OPN group, urinary leakage was observed and treated using ureteral stents in three patients, of which one patient continued to have urinary leakage despite stent insertion and was eventually treated with percutaneous nephrostomy.

In the LPN group, two patients required transfusion owing to low postoperative hemoglobin level. One patient had prolonged ileus, which resolved during the follow-up. Besides, two patients developed urinary leakage, of which the one with grade 1 leakage regressed during the follow-up, and the other one with grade 3a leakage was treated with a ureteral stent.

Pathological results were benign in three LPN (13.6%) and three OPN (7.3%) patients. Positive surgical margin was detected in two patients of each group (OPN: 4.8%, LPN: 9.1%). No recurrence was observed in these four patients.

Median follow-up time was 54 (37-78) and 62 (27-78) months for OPN and LPN groups, (p=0.471), respectively. The 5-year DFS rate was 92.7% (95% CI: 70.7-78.3) in the OPN and 95.5% (70.3-80.4) in the LPN group. No statistically significant intergroup difference was observed regarding DFS (log-rank=0.161 and p=0.688).

The 5-year CSS rate was 94.1% (95% CI: 74.1-78.5) in the OPN and 95.5% (71.2-80.1) in the LPN group with no statistically significant intergroup difference (log-rank=0.001 and p=0.987).

**Figure 1:**
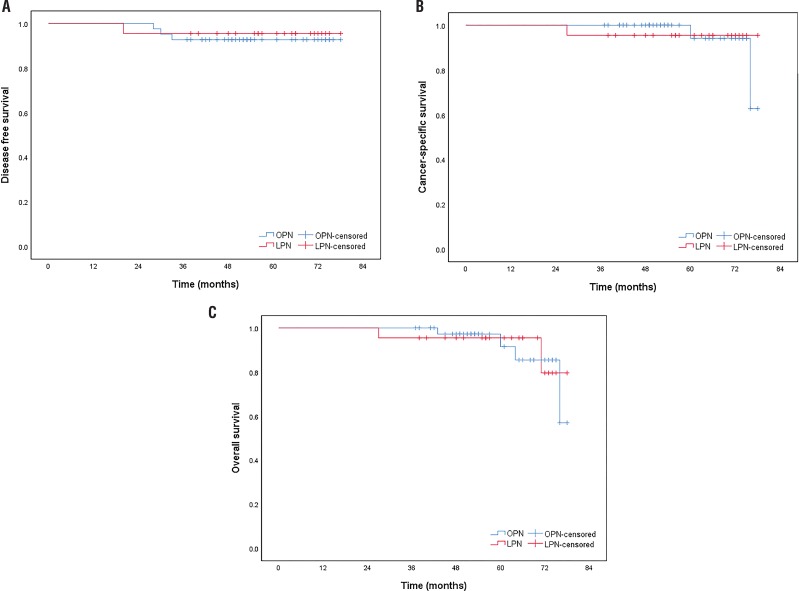
Disease-free survival (a), cancer-specific survival (b), overall survival (c) according to the surgical techniques.

The 5-year OS rate was 91.5% (95% CI: 71.5-77.6) in the OPN and 95.5% (69.8-79.3) in the LPN group with no statistically significant intergroup difference (log-rank=0.013 and p=0.909, Figure-1).

Two patients in the OPN and one patient in the LPN group relapsed, and all three died. The two patients of the OPN group died because of cardiac disorders after the fifth postoperative year, and the one patient from the LPN group with multiple comorbidities also died because of cardiac disease.

The univariate statistical analysis showed no significant difference between tumor size, surgical procedure, pathology, Fuhrman nuclear grade, R.E.N.A.L. score, and DFS (p>0.05). However, with the univariate analysis, we determined that the rate of recurrence increased significantly with age (RR=1.108, 95% CI: 1.006-1.220 and p=0.037). Furthermore, multivariate Cox proportional hazard regression analysis revealed that age was effective on the DFS irrespective of other factors. Each 10-year increase in age caused a statistically significant increase (2.891 times) in the development of recurrence regardless of other factors (95% CI: 1.010–8.254, Table-3).

**Table 3:** Results of the univariate and multivariate Cox regression analysis of the factors that may affect the disease-free survival.

	Univariate			Multivariate		
	RR	95% CI	p-value	RR	95% CI	p-value
**Age**	1.108	1.006-1.220	**0.037**	1.112	1.001-1.235	**0.049**
**Tumor size**	1.019	0.921-1.129	0.711	1.023	0.912-1.149	0.694
**LPN**	0.690	0.066-6.070	0.690	0.812	0.069-9.614	0.869
**Non-clear cell Ca**	$1,158	0.120-11.132	0.899	1.349	0.130-13.960	0.802
**Fuhrman grade**	1.968	0.305-12.689	0.476	1.527	0.180-12.991	0.698
**R.E.N.A.L. score**	0.712	0.300-1.691	0.441	0.666	0.210-2.109	0.490

**RR** = Relative Risk; **CI** = Confidence Interval; **LPN** = Laparoscopic partial nephrectomy; **Ca** = Carcinoma

The intergroup differences regarding mean GFR measurements, according to Bonferroni correction, were considered significant with p<0.0125, within the follow-up period. No significant differences were observed between the groups regarding GFR levels in the preoperative, first postoperative day, the sixth month, and the last visit, although GFR had an elevated course in the LPN group (p>0.0125).

The ΔGFR on the first postoperative day, sixth month, and last visit compared with the preoperative level was considered significant with p<0.0083 per the Bonferroni correction. No significant intergroup difference was observed regarding the first day, the sixth month, and last visit ΔGFR level compared with the preoperative level per the Bonferroni correction (p>0.0083; Table-4).

**Table 4:** Effects of open and laparoscopic partial nephrectomy on renal function

		OPN (n = 41)	LPN (n = 22)	p-value^a^
**GFR measurements**				
	Preoperative	83.71 ± 23.41	95.67 ± 28.20	0.077^b^
	Postoperative 1st day	81.15 ± 25.33	89.49 ± 29.11	0.241^b^
	Postoperative 6th month	72.49 ± 22.91	86.68 ± 27.50	0.033^b^
	Final control	67.51 ± 23.49	84.18 ± 26.92	0.013^b^
**ΔGFR**				
	Postoperative 1st day	−2.56 ± 12.08	−6.18 ± 10.99	0.248^c^
	Postoperative 6th month	−11.22 ± 9.68	−8.99 ± 5.89	0.329^c^
	Final control*	−16.20 ± 11.23	−11.49 ± 5.80	0.032^c^

* = Average follow-up period=4.8 years;

**OPN** = Open partial nephrectomy; **LPN** = Laparoscopic partial nephrectomy; **GFR** = Glomerular filtration rate; Δ **GFR** = Change in glomerular filtration rate, MDRD GFR (mL/min/1.73 m2);

a = Student's t-test;

b = According to the Bonferroni Correction, a p value less than 0.0125 was considered as statistically significant;

c = According to the Bonferroni Correction, a p value less than 0.0083 was considered as statistically significant.

All potential factors (age, ASA, ischemia time, surgical procedure)-considered predictive for ΔGFR on the first postoperative day compared with the preoperative period-did not have any significant positive predictive value (p>0.05).

In addition, none of the factors mentioned above had any significant predictive value in the sixth postoperative month (p>0.05). After the correction according to other factors, we determined that prolonged ischemia time caused a significant decrease in GFR level in the sixth postoperative month (B=-0.297, 95% CI: -0.558-0.036 and p=0.026).

All the potential factors thought to be effective on the prediction of ΔGFR were found to have no positive predictive value (p>0.05) at the last follow-up visit. We concluded that only the duration of the ischemia might have some effect on the sixth postoperative month (Table-5). One patient who underwent OPN with a preoperative GFR of 56mL/min/1.73m^2^ and had concomitant diabetes mellitus required hemodialysis in the fourth postoperative year.

**Table 5:** Multivariate linear regression analysis of potential predictive factors, which may affect the decrease in postoperative MDRD GFR

		Coefficient of regression (B)	95% CI for B		p-value
			Lower limit	Upper limit	
**Δ GFR 1st day**					
	Age	−0.139	−0.457	0.179	0.386
	ASA	−2.624	−7.640	2.392	0.299
	Ischemia time	−0.209	−0.573	0.155	0.255
	OPN	3.999	−3.273	11.270	0.276
**Δ GFR 6th month**					
	Age	−0.089	−0.317	0.139	0.436
	ASA	−1565	−5.158	2.028	0.387
	Ischemia time	−0.297	−0.558	−0.036	**0.026**
	OPN	−3.487	−8.696	1.721	0.185
**Δ GFR final control**					
	Age	−0.051	−0.311	0.209	0.695
	ASA	−2.602	−6.695	1.490	0.208
	Ischemia time	−0.296	−0.593	0.001	0.051
	OPN	−5.585	−11.518	0.348	0.065

* = Average follow-up period: 4.8 years;

**CI** = Confidence Interval; **ASA** = American Society of Anesthesiologist; **GFR** = Glomerular Filtration Rate; **CI GFR** = Change in Glomerular filtration rate; **OPN** = Open Partial Nephrectomy.

## DISCUSSION

Several studies have focused on the comparison of the long-term results of LPN and OPN in all T1 patients without particularly distinguishing between T1a or T1b. However, to the best of our knowledge, no other study has focused on the comparison of OPN and conventional LPN in T1b tumors. The studies that reported LPN results in T1b patients were typically comparing the results of the laparoscopic procedures in T1a patients (10-12).

Although LPN provides satisfying oncological results in tumors larger than 4cm per the report of Rais-Bahrami et al., the complication rate was higher and the hospitalization time was longer in the T1a group in this study (10). Rezaetalab et al. reported that patient satisfaction was higher, and narcotic analgesia requirement was lower in patients who underwent LPN (13). However, Becker et al. emphasized on the improved recovery time with LPN in T1 tumors and reported that there was no difference between OPN and LPN regarding the perioperative complication rates and long-term quality of life parameters (14). Our results demonstrated that laparoscopy was advantageous in nephron-sparing surgery. Although the complication rate was higher in our LPN group, the major complications were common in OPN group. Studies on PN have indicated shorter hospital stay for patients undergoing laparoscopy (15, 16). However, the reasons for not finding any differences in hospital stay between OPN and LPN in this study are probably because of our vast experience in OPN and slightly higher complication rates in LPN. Moreover, the tumors examined in previous studies were smaller in size, but our study included cases like T1b, wherein LPN was more challenging, which may have led to prolonged hospital stay in LPN cases compared with studies involving smaller-size tumors. Nonetheless, we believe that the length of hospital stay in LPN can be shortened with an increase in experience and appropriate patient selection.

However, LPN is known to be a relatively more difficult technique (17, 18). Despite three experienced endourologists in our clinic performing LPN, WIT, operation duration, and estimated blood loss were better in patients who underwent OPN, which was performed by several surgeons with different experience levels. Despite its technical difficulty, LPN has several advantages like reducing venous bleeding owing to pneumoperitoneum, providing better suturing under vision magnification, and facilitating the coagulation of small vessels (19). Marszalek et al. reported the opposite results and stated that WIT was shorter in LPN compared with OPN (16). The perioperative success with LPN solely depends on the surgeon’s experience. Although it could be expected that LPN might be beneficial regarding WIT because of pneumoperitoneum, WIT was longer in the LPN than the OPN group in our study. Notably, some studies have reported shorter WIT in LPN. Nevertheless, we believe that our conflicting results are based on the larger tumor sizes and higher mean R.E.N.A.L. scores. Also, theoretically, the surgical approach is generally more difficult, as tumors with a size between 4 and 7 cm may be more complicated and centrally located. A study by Simmons et al. reported that the transperitoneal approach was preferred mostly in stage T1b owing to the large tumor size and the need for pelvicalyceal repair and the rate of heminephrectomy was increased. Despite this, no difference was observed regarding intraoperative and perioperative complications compared with the smaller tumor (12). We obtained similar results in our study, and we observed only increases in the estimated blood loss and operation duration. If ever there was a significant difference in these values, it was not reflected in the complication rates.

Although the significance of SMP in the nephron-sparing surgery is still under debate, an increase may be seen in the LPN group because of the difficulty of the technique. Although it was somewhat difficult to assess SMP in our LPN group because of the small subject size, the SMP rate was higher in this group. The clear cell carcinoma evaluation of two patients (9.1%), who underwent LPN, displayed SMP but no recurrence was observed in these two patients during the 5-year follow-up period. In a recently published study, the investigators did not find a difference regarding SMP between laparoscopic, robot-assisted, and open techniques carried out in patients with T1b and T2a tumors, and they also reported that the stage of the tumor did not have any effect on SMP (20).

Several studies have reported low morbidity rates, suitable cost-effectiveness, and satisfying oncological results for laparoscopic PN (15, 21, 22). In the study conducted by Springer et al., OPN and LPN were performed in the treatment of T1 tumors, and the 5-year OS and CSS rates were 92% versus 94% and 88% versus 91% respectively (23). Lane et al. conducted a study focused on T1 tumors, and the separate evaluation of the T1b tumor showed that the 10-year DFS rate was 90% in patients who underwent LPN (24). In another study, 46 T1b patients were operated using robot-assisted LPN and the OS, DFS, and CSS rates were 97.1%, 97.1%, and 100%, respectively, after a 24.3-month follow-up (25). In our study, after a mean follow-up period of 58.1 [37-78] months, we concluded that OPN and LPN provided comparable oncological outcomes.

The oncological and functional outcomes should be reviewed during the selection of the surgical method for PN considering the increase in morbidity as a result of the decrease in renal functions. Despite the literature reporting that renal function impairment started with lesser than 20 minutes WIT and our intergroup difference regarding WIT, no difference was observed between short-term and long-term Δ GFR values (26). In our study, the analysis of the factors that might affect ΔGFR in long-term showed that only ischemia time was effective.

The limitations of our study were the retrospective design, small subject size, and the single-center outcome analysis. Moreover, LPN was performed only in selected patients owing to its implementation difficulty. We acknowledge the fact that drawing a meaningful comparison is difficult in such a small cohort. Although our clinic is a tertiary center, performing LPN and collecting more patients is difficult because of the challenging nature of LPN for tumors >4cm. We believe that our data could add up to the available literature and contribute to designing a meta-analysis.

The number of LPN is increasing with the increase in our experience, and we may be able to conduct randomized-controlled studies in the future. Patient data were collected prospectively in a newly designed database, with the plan of publishing the 10-year results. Further prospective, randomized, and controlled studies are needed to confirm that LPN provides oncological and functional outcomes similar to OPN in T1b tumors and it can be safely performed in T1b tumors because of the beneficial perioperative morbidity rates.

## CONCLUSIONS

Treatment of T1b RCC with OPN and LPN provide similar oncological and functional results in the long term. Nevertheless, more minor complications are observed in patients who underwent LPN. Technological advancement and experience have made LPN advantageous in terms of short hospitalization time and faster recovery process compared with OPN. However, considering its technical difficulty, LPN should be performed only in selected patients at experienced and high-capacity health centers.
